# Chondro-Osseous Respiratory Epithelial Adenomatoid Hamartoma of the Skull Base: Report of a Case and Literature Review

**DOI:** 10.1055/a-2556-0732

**Published:** 2025-04-03

**Authors:** Monica S. Trent, Beverly Wang, Edward C. Kuan

**Affiliations:** 1 Department of Otolaryngology – Head and Neck Surgery, University of California, Irvine Medical Center, Orange, California, United States; 2 Department of Anatomic and Clinical Pathology, Cytopathology, University of California, Irvine Medical Center, Orange, California, United States

**Keywords:** COREAH, chondro-osseous respiratory epithelial adenomatoid hamartoma, sinonasal mass

## Abstract

**Objective:**

Chondro-osseous respiratory epithelial adenomatoid hamartoma (COREAH) is a rare benign growth within the nasal cavity or paranasal sinuses. We aim to highlight the pathogenesis and clinical presentation of an extremely rare benign mass within the nasal cavity and demonstrate the workup, diagnosis, and management of this rare lesion, as well as review the literature.

**Methods:**

Retrospective review of a patient presenting with COREAH of the sinonasal cavity treated at a single tertiary academic medical center. The MEDLINE database was additionally searched for all case series or reports of sinonasal or skull base COREAH.

**Results:**

A total of sixteen articles were identified for review. In addition to the current case, a total of nineteen patients were identified in literature found to have sinonasal or skull base COREAH. The most common primary sites of attachment were the lateral nasal wall and posterior septum. Only two cases were identified with skull base attachment. Computed topography (CT) was employed in 84.2% of patients, and CT with MRI was utilized in 26.3% of patients. Endoscopic resection was performed in 100% of patients, with one recurrence reported in the literature and one incomplete resection requiring revision surgery.

**Conclusion:**

COREAH is an extremely rare hamartoma consisting of glandular proliferation with cartilaginous and osseous stroma. COREAH is symptomatic in 100% of reported cases in the literature, and complete surgical resection is often curative. Our case represents the longest follow-up reported with 4 years postoperatively revealing no growth or recurrence of COREAH.

## Introduction

A hamartoma is a benign mass consisting of mesenchymal tissues. These growths can arise in virtually every organ in the body and are most commonly found within the lungs, kidneys, and intestines. 1 They are characterized by their unique ability to create a disorganized proliferation of cells reflecting mature organ tissue structure unique from the native cellular architecture of the organ from which it arises. 2 Hamartomas differ from neoplasms due to their self-limited growth within the organ itself and lack of spread to distant sites. 3 It is important to distinguish between a hamartoma and a tumor as proper identification provides crucial information about management, including the avoidance of unnecessary risk of morbidity and the extent of surgical management. Furthermore, distinguishing hamartomas as the primary diagnosis is crucial for the early identification of syndromic conditions such as tuberous sclerosis, neurofibromatosis, or Peutz–Jeghers syndrome.

Although many hamartomas are found in association with syndromic conditions, it is more common they arise sporadically. They are categorized by the primary tissues from which they develop and are classified according to the predominant tissue type. 3 Those that develop within the nasal cavity are most commonly composed of respiratory epithelia, and therefore, the most common hamartomas found within the nasal cavity are respiratory epithelial adenomatoid hamartomas (REAH). There exists an extremely rare subset of REAH identified as chondro-osseous REAH (COREAH), which comprises both cartilaginous and bony tissues in conjunction with respiratory epithelium.

Prior reports of COREAH originating from the nasal cavity, paranasal sinuses, or skull base have been limited to case reports. 4 5 6 7 8 9 10 11 12 13 14 15 16 17 18 In addition to the current case study, one previously reported case in the literature demonstrated an attachment site along the skull base. 19 We present all previously reported cases of sinonasal and skull base COREAH in addition to one additional account of a young patient who presented with complete bilateral nasal obstruction and was subsequently found to have COREAH as well as ipsilateral ethmoid osteoma. In this way, we aim to analyze the current literature and review the methods of diagnosis, management, and treatment options for this benign tumor.

## Patients and Methods

### Study Design and Literature Review

This study was approved by the Institutional Review Board of the University of California, Irvine. A retrospective chart review of patients with COREAH of the paranasal sinuses and skull base treated at a tertiary academic medical center was performed. One patient was identified and the subsequent case was presented.

A comprehensive MEDLINE database search was performed between inception and 2024 for all cases of sinonasal and skull base COREAH. Search queries included “COREAH,” “chondro-osseous respiratory epithelial adenomatoid hamartoma,” “chondroosseous respiratory adenomatoid hamartoma,” “respiratory adenomatoid hamartoma” with “skull base,” “nasal cavity,” and “paranasal sinuses” specified and filters provided for human subjects. Within each case report, histology was obtained and confirmed to detail a diagnosis of COREAH. Information on age, gender, presenting signs and symptoms, attachment site, laterality, imaging modality, additional sites of involvement, number of open and transnasal endoscopic procedures, recurrence, and clinical outcome were extracted ( Table 1 ).

**Table 1:** Literature representation of COREAH

Study	Case #	Age (y)	Gender	Laterality	Attachment site	Symptoms	Duration of symptoms (mo)	Imaging modality	In-office biopsy vs. intraoperative biopsy	Clinical outcome (recurrence)	Follow-up (mo)
Flavin et al 4	1	11	M	U	Lateral nasal wall, middle turbinate	Nasal obstruction	6	CT, MRI	OR	None	6
Fang et al 5	2	3	M	U	Nasal roof	Nasal obstruction	NS	CT, MRI	IO	None	6
Nomura et al 6	3	7	F	B	Superior turbinate	Nasal obstruction	18	NS	NS	Recurrence after 12 mo, required second surgery	NS
Peric et al 7	4	68	F	B	Posterior middle turbinate	Nasal obstruction, mucopurulent rhinorrhea, postnasal drainage, snoring	24	CT	OR	None	12
Li et al 8	5	49	F	U	Superior turbinate	Nasal congestion, nasal obstruction, headache, facial pain, and pressure	36	CT	OR	None	3
Roffman et al 9	6	59	M	U	Posterior nasal septum	Nasal obstruction, headaches	36	CT	OR	Partially resected, 12 mo later definitive excision	12
Chatzopoulos et al 19	7	64	F	NS	Olfactory cleft	Nasal obstruction	12	CT	OR	None	12
Daniel et al 10	8	83	F	U	Posterior nasal septum	Headaches, perioral paranesthesia	48	CT, MRI	NS	None	6
Yu et al 11	9	54	F	U	Sphenoethmoidal recess	Nasal obstruction, hyposmia, rhinorrhea	NS	CT	OR	None	12
	10	57	M	U	Superior turbinate	Nasal obstruction, bloody mucopurulence	6	CT	IO	None	8
Nikolopoulos et al 12	11	66	F	U	Middle turbinate	Nasal obstruction, midface pain, headache	36	CT, MRI	OR	NS	NS
Fedda et al 13	12	38	F	U	Lateral nasal wall	Nasal obstruction	NS	CT	NS	None	NS
Nayani et al 14	13	55	F	U	Lateral nasal wall	Nasal obstruction, rhinorrhea, smell disturbances, epistaxis	48	CT	OR	None	6
Temmermand et al 15	14	75	M	U	Anterior–inferior nasal septum	Epistaxis	NS	NS	OR	NS	NS
Choi et al 16	15	34	F	U	Ethmoid	Nasal congestion, facial pain, hyposmia, poor sense of taste	NS	CT, MRI	IO	None	9
Idris et al 17	16	46	F	U	Lateral nasal wall	Nasal obstruction, rhinorrhea, anosmia	36	CT	OR	NS	NS
Beattie et al 18	17	31	M	U	Middle turbinate	Nasal obstruction, sore throat, odynophagia, pharyngitis, epistaxis	0.75	NS	NS	NS	NS
	18	60	M	U	Lateral nasal wall	Nasal congestion, facial pressure, rhinorrhea	NS	CT	NS	NS	NS
This work	19	18	M	U	Middle turbinate	Nasal obstruction, facial pressure, hyposmia, and thick nasal discharge	18	CT	OR	None	48

Abbreviations: B, bilateral; COREAH, chondro-osseous respiratory epithelial adenomatoid hamartoma; CT, computed tomography; F, female; IO, in-office; M, male; MRI, magnetic resonance imaging; NS, not specified; OR, intraoperative; U, unilateral.

### Case Presentation

This case is of an 18-year-old male without significant past medical history who presented with a 1.5-year history of complete bilateral nasal obstruction. He endorsed right-sided forehead and cheek pressure, bilateral nasal obstruction, hyposmia, and thick nasal discharge. Prior treatment attempts included two courses of antibiotics, one course of prednisone, and an unsuccessful attempt to utilize steroid nasal spray due to complete nasal obstruction. At our tertiary care center, nasal endoscopy established the presence of a large, bony polypoidal mass that completely obstructed the right nasal cavity, wrapped around the choana, and extended into the left nasopharynx. It was visualized despite thick white mucus along the floor of the nasal cavity. Additionally, there was severe polyposis involving all sinuses.

**Fig. 1:**
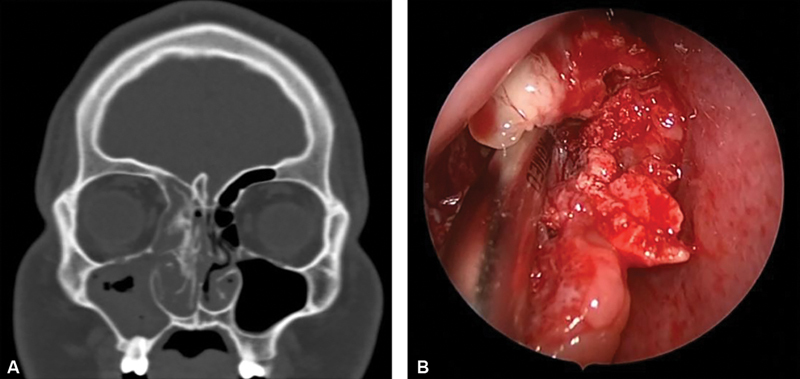
( **A** ) Computed tomography image of the patient prior to surgery, demonstrating fully opacified paranasal sinuses. ( **B** ) Intraoperative endoscopic endonasal image of the mass within the left nasopharynx.

Computed tomography (CT) of the paranasal sinuses determined the extent of the osseous obstruction. CT demonstrated right-sided pansinusitis with a hyperostotic right middle turbinate along with osteogenesis, indicating that the lesion appeared to originate from the vertical part of the right middle turbinate and was directly attached to the skull base ( Fig. 1A ). Additionally, CT incidentally identified a left-sided 5 mm ethmoid osteoma.

**Fig. 2:**
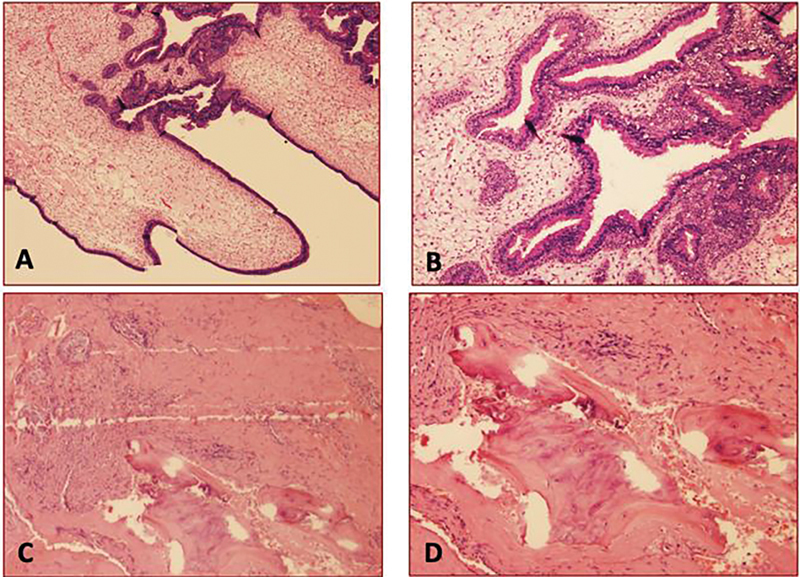
Sinus polypoid respiratory epithelial adenomatoid hamartoma (REAH) showing edematous stroma, with prominent glandular proliferation ( **A** ); closer view showing those glandular structures vary in size ( **B** ); areas of glands admixed with cartilaginous tissue ( **C** ); with osseous formation ( **D** ). Features are consistent with chondro-osseous REAH.

Informed consent was obtained for elective endoscopic surgical resection of the mass ( Fig. 1B ). Histological analysis intraoperatively confirmed the diagnosis of COREAH ( Fig. 2 ). Repeat CT at 15 months postoperatively demonstrated mild to moderate right frontal and ethmoid edema with no evidence of COREAH recurrence. Postoperative recommendations included utilizing steroid rinses directed within the nasal cavity twice daily for 3 months, then as needed for symptomatic management. The patient continues to remain asymptomatic with excellent nasal breathing, no facial pressure, and with intact sense of smell.

## Results

The Medline database was searched between inception and 2024, with the search strategy designed to identify all reports of COREAH originating in the nasal cavity, paranasal sinuses, or skull base. When the search was narrowed from REAH to COREAH, the number of articles reduced from 137 to 16. All identified studies were classified as case reports, with one as a poster presentation and two of which also provided a literature review from articles. The earliest report of COREAH was from 2004 and the literature publications range between 2004 and 2024. Including the single case from this case report, a total of 18 patients were included in this review. ( Table 1 )

Among the cases, the mean age at diagnosis was 46.2 years, with the ranges between 3 and 83 years. The primary sites were most frequently found along the lateral nasal wall, middle turbinate, superior turbinate, and nasal septum. Two patients (10.5%) had attachments along the skull base, including the patient in this reported study. All patients underwent endoscopic resection with histologically proven COREAH. Fourteen cases reported recurrence rates, with only one recurrence (7.1%) after 1 year of follow-up, and with one revision surgery (7.1%) after incomplete resection was performed. Twelve (85.7%) patients had no recurrence on follow-up, with the mean time of follow-up being 11.7 months and range between 3 and 48 months.

## Discussion

A hamartoma is a benign mass of mixed mesenchymal tissues arising either during embryonic germinal layer development or via sporadic proliferation. 3 Hamartomas can be classified depending on the primary tissue they are composed of. These distinctions are made depending on whether they are predominantly comprised of bone, cartilage, fiber, or nonmatrix tissues. 3 Nasal hamartomas have been occasionally described in the literature, the most common of these being REAH. Another rare lesion described in the literature is nasal chondromesenchymal hamartoma (NCMH). COREAH are, in contrast, exceedingly rare. To the best of our knowledge, only 18 patient cases of COREAH have been reported in the literature to date. 4 5 6 7 8 9 10 11 12 13 14 15 16 17 18 COREAH differs from other hamartomas histologically as it consists of both mesenchymal and epithelial tissues. Unlike REAH, which is composed of only respiratory epithelium, and NCMH, which is composed of cartilaginous tissues, COREAH is unique because it consists of respiratory epithelium-lined glands, cartilaginous tissues, and osseous stroma. 3 7 21 COREAH has been diagnosed in a wide range of patients, whereas REAH typically affects predominately male patients in their third to ninth decade of life, and NCMH is predominantly found in individuals younger than 3 months and is associated with a DICER1 genetic mutation. 10 20 22 23

The pathogenesis of COREAH is still largely undetermined. A formal literature review reveals that it has affected patients ranging from the ages of 3 to 83 years, which makes it highly unlikely that COREAH results only from congenital overgrowth of the germinal matrix. 10 We must assume, therefore, that like other hamartomas, COREAH can arise spontaneously as we age and is likely acquired. Because COREAH is related to REAH, much can be understood with an understanding of the pathogenesis of REAH. Similar to COREAH, REAH typically presents with symptoms of nasal obstruction, deviated septum, sinusitis and rhinorrhea, repeat sinus surgery, hyposmia, and headache, and is largely associated with chronic inflammation of the nasal passages and nasal polyps. 13 23 24 25 Because REAH commonly occurs in concert with inflammatory process it has been suggested to be due to a chronic reaction induced by inflammation. 25 Tryptase-producing mast cells have been implicated in the inflammatory component that may be contributing to REAH growth. 26 It is possible that many of these features of REAH also pertain to COREAH, though there is much more to be discovered about this rare tumor. Despite the modest information on COREAH genesis, it is widely accepted that endoscopic resection, whenever possible, is the most favorable option for successful treatment. 27 28 29

Prior to surgery, COREAH must be distinguished clinically and histologically from inflammatory polyps, inverted papilloma, and sinonasal adenocarcinoma to ensure effective treatment. 10 23 Obtaining the correct pathological diagnosis is particularly important in COREAH because it is so rare and can be mistaken for a neoplasm. Overtreatment is a risk in the management of these lesions and with the correct diagnosis, surgeons will successfully avoid over-treating these benign hamartomas with endoscopic craniofacial resection.

COREAH can be differentiated from other diagnoses through its clinical and histological presentation. A CT scan is a valuable tool in distinguishing regional predilections of COREAH, and contrasting it with regions more commonly afflicted by benign and malignant sinonasal and skull base lesions. Clinically, COREAH tends to present unilaterally and will affect the posterior nasal septum more often than inflammatory polyps do. 23 24 27 COREAH can also be distinguished from a diagnosis of sinonasal adenocarcinoma because sinonasal adenocarcinoma typically involves the middle turbinate or will often develop within the ethmoid sinus. 23 In contrast to COREAH, inverted papilloma will frequently involve the lateral nasal wall and present with aggressive areas of bone erosion. 8 13 Inverted papilloma also have the capacity for malignant transformation, thus it is especially important to distinguish this from a benign COREAH. 13 23 30 Histologically, COREAH consists of respiratory epithelium along with an abundance of chondro-osseous stroma. 3 7 21 This is distinguished from nasal polyposis, which presents as seromucinous glandular, edematous tissue. 13 23 Sinonasal adenocarcinoma is highlighted by its cribriform architecture composed of ciliated epithelium and glandular tissue, and inverted papilloma consists of stratified squamous epithelium mixed with mucin and microcysts. 23 24

Once correctly identified, COREAH may be managed with endoscopic resection and appears to have favorable outcomes overall. COREAH has only one reported recurrence in the literature, and only 4.1% of patients with REAH have been reported to show recurrence after endoscopic resection. 6 28 However, long-term surveillance and monitoring, as well as noting changes in symptoms, remains important.

## Conclusion

COREAH is an extremely rare hamartoma consisting of prominent glandular proliferation separated by cartilaginous and osseous stroma. COREAH is symptomatic in 100% of reported cases in the literature, and complete surgical resection of COREAH is curative in the majority of patients, including the patient we present in this report.
